# Pollen wall patterns as a model for biological self‐assembly

**DOI:** 10.1002/jez.b.23005

**Published:** 2020-09-29

**Authors:** Asja Radja

**Affiliations:** ^1^ School of Engineering and Applied Sciences Harvard University Cambridge Massachusetts USA

**Keywords:** exine, exine pattern, mathematical modeling, morphometrics, pattern formation, physical modeling, pollen, self‐assembly

## Abstract

We are still far from being able to predict organisms' shapes purely from their genetic codes. While it is imperative to identify which encoded macromolecules contribute to a phenotype, determining how macromolecules self‐assemble independently of the genetic code may be equally crucial for understanding shape development. Pollen grains are typically single‐celled microgametophytes that have decorated walls of various shapes and patterns. The accumulation of morphological data and a comprehensive understanding of the wall development makes this system ripe for mathematical and physical modeling. Therefore, pollen walls are an excellent system for identifying both the genetic products and the physical processes that result in a huge diversity of extracellular morphologies. In this piece, I highlight the current understanding of pollen wall biology relevant for quantification studies and enumerate the modellable aspects of pollen wall patterning and specific approaches that one may take to elucidate how pollen grains build their beautifully patterned walls.

## Research Highlights

The patterned pollen surface is an ideal system for uncovering pattern formation mechanisms. Relevant developmental steps and genes involved in pollen pattern formation are summarized, and mathematical and physical applications are proposed.

## 1 INTRODUCTION

The ability to predict an organism's form from its genetic code still eludes us in most systems. Part of the difficulty in creating such a mapping is that the products of the genetic code (macromolecules) are under the influence of physical forces that are independent of genetically encoded information. Therefore, understanding how biological shapes develop depends not only on identifying these products, but also on understanding how these building blocks physicochemically interact with each other independently of the genetic code. A complete understanding of how biological forms develop requires a coupling of molecular and physical mechanisms over many length and time scales.

Thompson (1917) was one of the first scientists to quantify natural shapes and consider how physical forces influence their form during development and throughout evolution. Although his geometric and physical approaches in *On Growth and Form* are modest, his work was pivotal in its insights and breadth of new quantitative approaches for describing biological shapes. In the past several decades, there has been a growing interest in understanding how physical mechanisms, often originally used to describe nonliving systems, influence the development of biological shapes and patterns. The number of mathematical and physical frameworks applied to morphology and pattern formation across many length and time scales has been growing at an impressive rate. Morphometric shape analysis, reaction–diffusion approaches, and mean‐field theory methods are all examples of such frameworks used to quantify emergent shapes—from constituent polymers to groups of cells to whole tissues and even collections of organisms. More recently, there has also been a push to discover potentially new unknown physical strategies that nature has harnessed to create shapes and patterns. These “Rules of Life” will be directly motivated by and tailored to understand complex biological systems.

**Figure 1:**
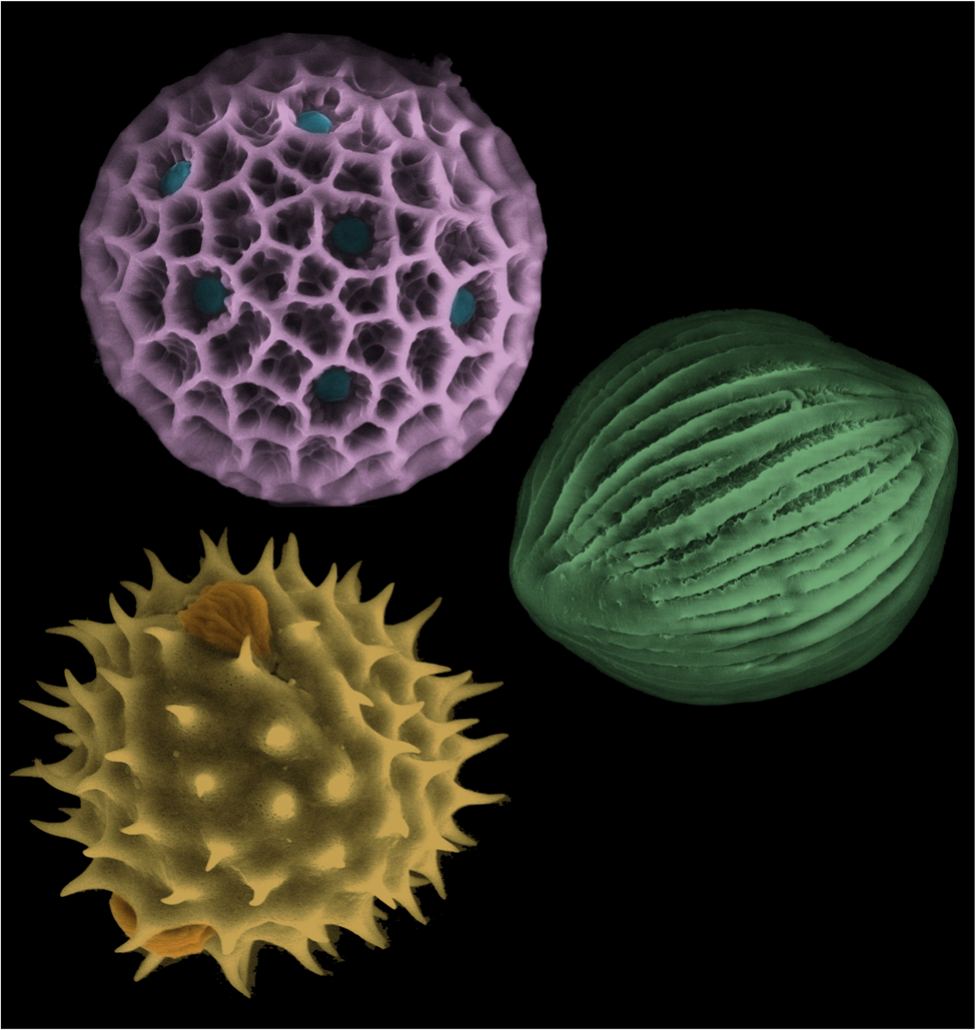
The diversity of pollen patterns. The figure presents three pollen grain species with three different wall patterns. Clockwise starting from the upper left: *Phlox* sp. (hole‐y exine falsely colored purple, pore apertures falsely colored blue), *Spathiphyllum cannifolium* (stripped exine falsely colored green), and *Calendula* sp. (spiked exine falsely colored yellow, aperture bumps falsely colored orange). *Phlox* sp. aperture patterning fits with the exine patterning, *Spathiphyllum cannifolium* has no apertures, and *Calendula* sp. is a eudicot with a quintessential pattern of three apertures distributed on the cell equator [Color figure can be viewed at wileyonlinelibrary.com]

An understanding of how various patterns and shapes emerge in biological systems can be considered complete when both the genetic products and the physical rules that govern their interactions are identified. Theoretical frameworks that quantify elucidated physicochemical processes can then be related back to the genome by parametrizing such models with physicochemical properties of the genetic code products. Overall, this is a hugely complicated and ambitious task. A tangible starting point is with single‐cell morphologies. These systems have significantly fewer confounding variables and a smaller range of length scales than those dictating pattern formation in whole organisms. Furthermore, studying specifically pollen wall patterning, which is particularly diverse and species specific (Figure 1), will help us to discover some of the “Rules of Life” that dictate how extracellular patterns form. This will also add to our general understanding of the diversity of pattern formation mechanisms in biological systems. The micron‐sized patterning on a curved surface is not unique to pollen grains as similar polygonal, striped, and spiked patterns are found on curved surfaces across taxa from fungal spores and insect exoskeletons to coccoliths and radiolaria (Haeckel, 1998; Harrison & Locke, 1998; Kärik et al., 1983). The universality of such patterns further suggests that some pattern formation processes may not depend on the unique genetic codes or molecular mechanisms of each individual system but rather on common physical processes that shape the system's fundamental components.

The pollen cell wall is an ideal system for studying how physical pattern formation mechanisms can generate a variety of extracellular morphologies for three main reasons. (1) Pollen wall patterns are one of the most diversely patterned biological systems whose breadth is well‐documented in a publicly available centralized database, PalDat (Weber & Ulrich, 2017). (2) The cell wall developmental steps have been well‐documented for a large number of species using electron microscopy (EM; Blackmore et al., 2007; Quilichini et al., 2015; Scott, 1994). (3) There is a growing understanding of the genes and their products required for successful cell wall pattern formation. Yet, few quantitative studies explore morphometric representations or physicochemical pattern formation mechanisms of the pollen wall (Ariizumi & Toriyama, 2011; J. Shi et al., 2015; Wang & Dobritsa, 2018). The available data on pollen wall morphology and its development makes this system ripe for mathematical quantification, physical modeling, and theoretical studies, and, therefore, it is an excellent system for discovering some of the “Rules of Life” dictating single‐cell cuticle morphologies.

The structure and development of the multilayered pollen wall is exceptionally complicated and unique. Therefore, my goal is to highlight pertinent research in pollen wall form, function, and development in the “Biology of pollen grain wall” section such that it is accessible to researchers outside of the field who are interested in quantification and modeling. Note that this section is not biologically comprehensive; there are more detailed explorations of the current state of pollen wall research (Ariizumi & Toriyama, 2011; Blackmore et al., 2007; Quilichini et al., 2015; Wang & Dobritsa, 2018). In Section 3, I highlighted the few studies that quantify pollen patterns and mathematically model relevant physical processes, and I suggest improvement to these approaches. Most importantly, I specify the aspects of pollen wall patterning that can be modeled and the approaches that are the most appropriate. I hope this inspires others to explore the world of palynology as it provides an awe‐inspiring example of “morphogenesis in the miniature” waiting to be revealed (Heslop‐Harrison, 1972).

## 2 BIOLOGY

### 2.1 Form

The pollen outermost layer features some of the most beautifully intricate and diverse patterns found in nature (Figure 1). They are spiky in daisies, have stripes in roses, and appear foam like in daffodils and jasmines. The wall morphology within a species is highly conserved, while patterns in different species greatly vary. Consequently, pollen are often used for fossil dating (Brown et al., 1989), forensic analysis (Horrocks et al., 1998; Milne et al., 2004), and climate change studies (Bartlein et al., 1998; Jackson et al., 2000).

**Figure 2:**
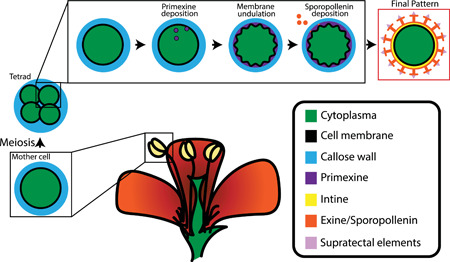
Schematic of pollen wall development. The above schematic represents the meiotic, tetrad, and final pattern stages of pollen grain wall development. Pollen grains develop inside the anther in a fluid environment, and each cell is encased in a callose wall. Following meiosis, they enter the tetrad stage during which time a transient fibrilar material, called the primexine, templates the cell surface. The underlying membrane undulates with a periodicity that is reinforced by sporopollenin deposition, and the final mature pattern reflects these initial undulations [Color figure can be viewed at wileyonlinelibrary.com]

Despite their huge morphological diversity, the multilayered pollen walls across all seed‐bearing plants are well‐conserved (Figure 2, red box labeled “Final Pattern”). The patterned outermost layer is called the *exine* (Erdtman, 1986; Figure 2, red box, orange layer). In some species, the cross‐section of this layer looks like a series of T‐shaped rods that extend radially outwards from the pollen surface. The stem portions that are perpendicular to the surface are called *baculae* and the roofs, which are sometimes connected, are called *tecta*. Different distributions and connections of these Ts make up the unique patterns on the surface. The exine is made of an extremely chemically robust material called *sporopollenin* whose composition has only recently been elucidated (F. Li et al., 2019). While there are other wall layers underneath the exine and decorations deposited on top of the exine, these are out of the scope of this paper.

Pollen surfaces can also be patterned with a varying number and geometric arrangement of *apertures*. Apertures are regions of reduced or absent exine through which the genetic material exits during germination (blue–green highlighted disks in the top left pollen grain and red–orange highlighted protrusions in bottom left pollen grain of Figure 1). Like exine patterning, aperture patterning is highly conserved within a species, and varies between species. Examples of aperture patterns include single‐pores, distributions of pores or bulges on the surface, and stripes around the pollen equator. Together, the exine and aperture patterning lead to the huge diversity of pollen patterns.

Palynologists have developed a system of terms that qualitatively describe exine and aperture patterns. Currently, this accepted terminology is quite extensive, making it somewhat inaccessible to nonspecialists, though several illustrated glossaries have attempted to mitigated this issue (Halbritter et al., 2018; Punt et al., 2007; Weber & Ulrich, 2017). For example, the term “reticulate” is used to describe foam‐like patterns, “echinate” for spikes, “striate” for stripes, and so on. These terms and corresponding images are carefully cataloged in paldat. org, an open‐source website that features over 27,000 EM images of almost 3200 species in over 1400 genera (constituting about 44% of present‐day seed‐producing families). Other databases for morphologically diverse systems including insect eggs (Church et al., 2019), pavement cells (Vöfély et al., 2019), fungal spores (Kärik et al., 1983), diatoms (Kociolek et al., 2020), and radiolaria (https://www.radiolaria.org) are also incredibly important because they lower the entry barrier for researchers external to the field to develop and test quantitative models.

### 2.2 Function

The pollen grain is a compact package for the plant's male genetic material. Its external exine wall is hypothesized to have several functions. The physically and chemically robust sporopollenin constituting the patterned surface protects the genetic material from external biotic and abiotic forces (such as microbial attack or UV radiation and high temperatures). While the functions of the wall material properties are well‐understood, the function of the pattern itself is not well‐elucidated. Some wall patterns may facilitate the efficient delivery of pollen between plants, but careful quantitative models are lacking. Several studies have found a correlation between pollinator type and surface pattern: wind‐ or water‐pollinated pollen often have smooth walls whereas pollen carried by animals often have patterned walls with projecting features that attach to the animal (Culley et al., 2002; Heslop‐Harrison, 1971; Sannier et al., 2009). However, again, models describing such fluid dynamics or physical interactions are needed. In addition, the wall patterning itself may contribute to species recognition once the pollen grain comes into contact with the female plant organ. Divots in the wall may act as containers for a material necessary for species recognition (Zinkl et al., 1999), and/or the surface pattern may physically complement the female receptor such that the two portions of a single species fit together (Lin et al., 2016). Finally, a recent quantitative macroevolutionary study organized all pollen surfaces into symmetric or disordered patterns and found that disordered patterns are more often evolutionarily selected for (Radja et al., 2019).

Apertures also perform several functions; they are the sites of water transport (Heslop‐Harrison, 1979; Vieira & Feijo, 2016) and where the genetic material breaks through the wall during germination (P. Li et al., 2018). Some studies found a general trend of increasing aperture number in flowering plants, which conceivably increases the likelihood of successful germination or water transport (Furness & Rudall, 2004; Heslop‐Harrison, 1979). Some aperture patterns have been quantitatively modeled to demonstrate that specific patterns provide energetically efficient folding pathways such that the pollen grain can desiccate upon leaving the anther and rehydrate upon contact with the female organ without breaking (Katifori et al., 2010). Finally, the overwhelming prevalence of a particular aperture pattern, three narrow stripes distributed perpendicularly around the pollen equator as in *Arabidopsis thaliana*, indicates that this configuration of apertures might be an optimal adaptation (Furness & Rudall, 2004; Prieu et al., 2016). While some preliminary experiments have identified that this configuration exhibits the highest resistance to osmotic stress (Albert et al., 2010; Prieu et al., 2016), mechanistic models do not exist. Lastly, while function often follows form, there is no current macroevolutionary consensus as to which features of pollen patterns may be evolutionarily selected for and why. It is entirely possible that some pollen patterns are the result of evolutionary drift of biophysical and/or biochemical processes.

### 2.3 Exine development

Our understanding of pollen wall development in seed‐bearing plants has been built over the last 40 plus years through careful morphological observations predominantly using transmission EM, an imaging technique that provides a cross‐sectional view of the developing subcellular features of the pollen wall (Owen & Makaroff, 1995; Paxson‐Sowders et al., 1997; Scott, 1994). All pollen grains develop in a fluid environment within a plant organ called the anther (Figure 2). Initially, each pollen cell is contained within a unique transient cell wall made of β‐1,3‐glucan, a sugar composed of glucose monomers, called the *callose wall* (Kauss, 1996). Following meiosis, the resulting daughter cells remain contained within this callose wall in a group called the *tetrad* such that they are isolated from both other tetrads and from each other within the tetrad (Nishikawa et al., 2005; Waterkeyn, 1962). Next, a crucial transient fibrillar matrix called the *primexine* is deposited on top of the plasma membrane (PM) and within the callose wall. Roughly at a similar time, the PM begins to undulate with a wavelength that is specific to each species. During the last few developmental steps, vertical rods called *probaculae* form on the peaks of these undulations (Xu et al., 2016) and act as a blueprint for the subsequent deposition of sporopollenin when the callose wall is degraded (Ariizumi & Toriyama, 2011; Fitzgerald & Knox, 1995). The deposition of sporopollenin onto probacula (and protecta) to form the baculae and tecta completes the development of the patterned exine.

The following genetic studies in the model organisms *Arabidopsis thaliana* (which produces a reticulate or foam‐like exine pattern) and *Oryza sativa* (which produces an “areolate” or bumpy exine pattern) have confirmed that successful wall patterning cannot be achieved without proper primexine construction, callose wall formation, and PM undulations. The pollen walls of mutants including defective in exine formation 1 (Ma et al., 2013; Paxson‐Sowders et al., 2001), no exine formation 1 (Ariizumi, Hatakeyama, Hinata, Inatsugi, et al., 2004), and no primexine and PM undulation (Chang et al., 2012) exhibit poorly formed primexine, a thin callose wall, and no PM undulations during development. As a result, they all have a poorly constructed mature exine and unviable pollen. Due to these studies, it is now assumed that exine pattern formation requires three components: the callose wall, primexine, and PM. Furthermore, it is hypothesized that the interactions between these three components dictate the wavelength of PM undulations, which, in turn, determines the wavelength, and thus the pattern of the fully developed exine. Interestingly, the exine formation defect mutant has normal PM undulations despite having a defective primexine during development (Hu et al., 2014). While it is possible that either the primexine was not well‐imaged in the study or it was well‐formed enough to induce PM undulations, more studies are needed to definitively identify why primexine is seemingly not required for PM undulations in this particular mutant. Finally, it is important to note that the functions of the proteins associated with the identified mutants above are unknown. As such, their influences could be structural, mechanistic, chemical, and/or physicochemical, and more studies are required to elucidate how they impact pattern formation.

### 2.4 Primexine development

Our understanding of primexine development and how it influences the final exine pattern is quite poor. Part of the problem is that the primexine is both ephemeral and difficult to image. Nevertheless, several studies have shown that the primexine structure changes during development. Early in the tetrad stage, “spacers” appear as electron‐dense portions of the primexine within the wells of PM undulations (Fitzgerald & Knox, 1995; Paxson‐Sowders et al., 2001; Quilichini et al., 2015). Later in development during probacula formation, various amphiphilic assemblies develop within the primexine. Both of these structural features have led to the current assumption that self‐assembly processes influence primexine development and the pattern formation of exine (Gabarayeva & Grigorjeva, 2016; Hemsley et al., 2003; Hemsley & Gabarayeva, 2007).

### 2.5 Primexine composition

The primexine composition has not been explicitly identified, however several studies employing various staining methods have revealed that the primexine in *Arabidopsis thaliana* pollen likely contains cellulose (Heslop‐Harrison, 1968), xylans (W. Li et al., 2017), arabinogalactan proteins (AGP), and pectins (Suzuki et al., 2017). Each of these components is localized in different regions of the primexine during development. It appears that pectins are confined to the troughs of PM undulations, whereas AGPs and xylans follow the reticulate patterning (i.e., they are found on the peaks of PM undulations during probacula formation; Suzuki et al., 2017). The primexine is also predicted to be composed of other plant polysaccharides, glycoproteins, and potentially lipopolysaccharides (Gabarayeva & Grigorjeva, 2016; Heslop‐Harrison, 1968). All of these constituents are likely synthesized in both the cytoplasm of individual developing daughter cells and the surrounding cells of the anther wall innermost layer (Ariizumi, Hatakeyama, Hinata, Sato, et al., 2004; Heslop‐Harrison, 1971; Rodriguez‐Garcia & Majewska‐Sawka, 1992; Rowley & Dunbar, 1970).

Careful imaging of wall development in knockout mutants involved in carbohydrate synthesis of primexine has revealed the potential role of various components in exine pattern formation. SPONGY2/irregular xylem‐9‐like (SPG2/IRX9L) encodes a presumed glycosyltransferase that synthesizes xylan in the primexine (Dobritsa et al., 2011; W. Li et al., 2017). The exine of these mature mutants appears bumpy, does not have tecta, and the baculae are distributed at a higher density on the surface. It is thought that this higher density results from an incorrectly formed primexine that looks very thin during early developmental and whose xylan distribution during probacula formation is highly irregular. While it is unknown whether PM undulations are disrupted, it is possible that xylan‐containing units in the primexine are required for the proper spacing of probaculae during development. The disruption of this process leads to a different PM undulation wavelength and a higher density of baculae. Nodulin intrinsic protein 7 is a known boric acid facilitator that plays a role in the rhamnogalacturonan II synthesis in pectic cell walls. While there are no careful imaging studies of this mutant's wall development, the mature walls have structural defects. Thus, it is possible that disruptions in pectic primexine components may affect the early stages of exine patterning (Routray et al., 2018). Unequal pattern of exine 1/kaonashi 4 encodes a glycosyltransferase that is likely involved in the biosynthesis of AGPs or of rhamnogalacturonan I in the primexine (Dobritsa et al., 2011; W. Li et al., 2017; Suzuki et al., 2017). Developmental studies of these mutants show that the deposition of an irregular primexine and diffuse probaculae lead to a patchy mature exine. Therefore, in combination with staining studies, it is hypothesized that primexine‐localized AGPs play a vital role in the early‐stage primexine structure and could function as a substrate for sporopollenin adhesion in the later tetrad stage. Like IRX9L/SPG2 mutants, kaonashi 2 (KNS2) mutant walls have a abnormally high density of baculae, and it is hypothesized that the KNS2 gene might also be involved in ensuring proper wavelength of PM undulations. KNS2 encodes a sucrose phosphate synthase linked to carbohydrate synthesis (Suzuki et al., 2008); however, it is unknown if the primexine, callose wall, or PM are affected in this mutant. Ruptured pollen grain 1 (RPG1/SWEET8) and thin exine 2 (TEX2) encode sugar transporters with unknown substrates that are likely involved in primexine synthesis. *rpg1/sweet8* mutants have partially or completely missing primexine and thus have completely defective mature walls, while *tex2* mutant walls are have randomly deposited baculae and seemingly no tecta (however, there are no developmental imaging studies of the primexine; Dobritsa et al., 2011; Guan et al., 2008; Sun et al., 2013). Finally, defective pollen wall 3 in rice may be involved in primexine transport since neither primexine matrix deposition nor PM undulations are observed during wall development in mutants (Mondol et al., 2020).

### 2.6 Callose wall deposition

The callose wall also influences pollen wall patterning. Callose synthase 5, glucan synthase‐like 1 (GSL1) and GSL12 in *Arabidopsis thaliana* (Dong et al., 2005; Nishikawa et al., 2005), and GSL5 in rice are required for callose wall formation (X. Shi et al., 2015). Knockouts of these genes have defective callose walls during development and an aberrant exine with random depositions of sporopollenin. Therefore, it is likely that proper callose wall formation is required for exine patterning. Interestingly, some aquatic plant pollen that have greatly reduced or entirely absent callose walls develop unpatterned mature exine (Pettitt, 1981; Takahashi, 1995).

### 2.7 Aperture development

During primexine deposition and PM undulation distinct regions of the PM remain in close contact with the callose wall, ostensibly delineating the regions of future apertures (Echlin & Godwin, 1969; Heslop‐Harrison, 1968; Wang & Dobritsa, 2018). A little is known about the molecular, cellular, and physical mechanisms specifying the position and number of these apertures. Correlations between aperture pattern and ploidy, cytokinesis, or mitotic spindle formation have been revealed; however, there are no mechanistic models that causally relate any of these features to aperture organization. For example, aperture number is proportional to ploidy, which increases as pollen size increases (Reeder et al., 2016). However, the biochemical or mechanical properties linked to ploidy (or cell size) and changes in aperture number are unknown. Similarly, some aperture arrangements reflect the packing geometry of pollen cells in the tetrad during development. It was previously thought that the points of last contact between cells after meiosis dictate future aperture positions (Wodehouse, 1935). Now it is considered unlikely that these points of last contact directly delineate aperture positions; however, the mechanisms for determining aperture position remain unknown (Zhou & Dobritsa, 2019). It is difficult to conceive how arrangements of six or more apertures form by this model of aperture development (Figure 1, *Phlox* sp.). It may be possible that different patterns form through a range of molecular and/or physical mechanisms. The cellular mechanisms that may play a role in aperture formation have been reviewed in previous publications (Wang & Dobritsa, 2018; Zhou & Dobritsa, 2019).

A handful of genetic studies have identified a few genes associated with aperture development in *Arabidopsis thaliana*. Inaperturate pollen 1 (INP1) was the first gene discovered to influence aperture formation (Dobritsa & Coerper, 2012; Dobritsa et al., 2011). The INP1 protein clumps into three perpendicular lines around the equator delimiting the future apertures of *Arabidopsis*; however, the exact function of this protein and how it influences aperture formation or placement is unknown (Dorbitsa et al., 2018; P. Li et al., 2018). More recently, a protein kinase called D6 protein kinase‐like 3 (D6PKL3) was discovered to presumably act upstream of INP1 and attract it to the proper aperture sites (Lee et al., 2018). While both INP1 and D6PKL3 are correlated to future aperture placement, it is unlikely that either determines aperture placement. Large and square pollen (*lsq*) mutants exhibit a higher number of aperture stripes in *Arabidopsis* mutants. While the specific molecular mechanism leading to this increase is unknown, it is presumed that the increase in aperture number is correlate to an increase in pollen size (Dobritsa et al., 2011). Two more mutants with defective apertures, macaron (*mcr*) and doughnut (*dnt*), were recently identified through a forward genetic screen, however their associated genes remain unknown. The *mcr* mutant has a ring‐like aperture that passes through the poles of the pollen grain, and the dnt mutation has two round hole‐like apertures at the poles of pollen grains (Plourde et al., 2019). More detailed information on mutants and the hypothesized molecular mechanisms of aperture formation is summarized in two recent reviews (Wang & Dobritsa, 2018; Zhou & Dobritsa, 2019).

## 3 MATHEMATICAL/PHYSICAL MODELING OF POLLEN GRAIN WALLS

### 3.1 Morphometrics

The rapid and accurate classification of the many pollen patterns both extant and in the fossil record is imperative for applied fields including vegetation reconstruction, climate change studies, allergy research, forensic science, oil exploration, and, of course, plant evolutionary studies and studies of pollen wall function. Currently, pollen taxonomic identification relies upon qualitative descriptions of pollen wall patterns (see section on “Form”). Consequently, similar pattern types are separated into categories that can be consistently classified by multiple analysts. This results in a coarse taxonomic resolution of the pollen wall morphospace. Image‐based algorithms, on the other hand, can rapidly distinguish subtle morphological differences between taxa that are easily observed by the human eye but are not easily conveyed by the existing qualitative terminology. There have been a few attempts to classify pollen wall patterns using state‐of‐the‐art approaches such as deep learning (Daood, Ribeiro, and Bush, 2016), supervised machine learning (Punyasena et al., 2012; Tcheng et al., 2016), and artificial neural networks (Holt et al., 2011; Kaya et al., 2013), often in concert with pattern recognition algorithms. While these automated approaches for classifying surface texture features are particularly useful for rapid and efficient identification crucial to many of the fields mentioned above, the features they identify are often not biologically relevant. Therefore, they are not ideal for evolutionary studies and understanding of wall patterning mechanisms where interpretability of the identified features is particularly important.

**Figure 3:**
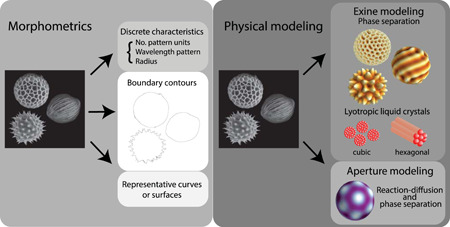
Flow diagram of quantification approaches. Morphometrics: One can represent the morphology of pollen as discrete characteristics, boundary counters, or representative curves or surfaces. Physical modeling: One can model the exine or aperture patterns. Different aspects of exine patterning can be recapitulated through phase separation modeling or using lyotropic liquid crystal systems. Both reaction–diffusion systems and phase separation models recapitulate the aperture patterning of different species [Color figure can be viewed at wileyonlinelibrary.com]

Low‐dimensional morphospaces parametrized by biologically relevant features allow us to make observations about the distribution of patterns that may reveal new hypotheses in evolutionary relationships and/or pattern formation mechanisms. Examples of morphospace parameters include measurements of pollen geometry, such as radius, pattern wavelength, and/or pattern feature width (i.e., the width of a spike; Figure 3). However, we lose imperative information about the whole pollen pattern when we limit its description to a few discrete characteristics, and morphospaces defined in such a way often have regions with overlapping species. While mathematical techniques such as principal component analysis, which identifies new parameters as linear combinations of uncorrelated measurements, result in more finely grained morphospaces (Arzani et al., 2005; Pardo et al., 2010; Ueda & Tomita, 1989), computational image analyses that quantify features such as complexity (Mander et al., 2013) or connectivity are better because they also generate biologically interpretable features. Other methods, such as landmark‐based shape analysis, which define shapes as a set of points samples along a boundary, and elliptical Fourier analysis, which decomposes closed outline curves into Fourier‐like modes (Bonhomme et al., 2013; Kuhl & Giardina, 1982), have also been used to quantify and classify pollen shapes (Figure 3).

The above methods typically use two‐dimensional (2D) representations of the surface, however pollen grains have a complicated 3D structure. Fortunately, 3D imaging methods are being developed (such as a label‐free technique of optical diffraction tomography (Kim et al., 2018) and superresolution microscopy methods (Sivaguru et al., 2018)) that provide data for quantification studies to most accurately describe the complete pollen surface morphology. Ideally, pollen grain surfaces could be represented as continuous intrinsic surfaces (Figure 3); however, this is difficult to implement since there are no canonical parametrizations that currently allow for the natural registration of two surfaces (Srivastava & Klassen, 2016). Two potentially useful methods to pursue are persistent homology (PH), a mathematical method that captures topological features across scales that has already been applied to quantify 3D structures such as teeth and plant roots (M. Li et al., 2017; Turner et al., 2014), and functional shape analysis (FSA), a method for understanding continuous shapes by developing statistical models for continuous functions and curves which are used to generate finite observations (Srivastava & Klassen, 2016). However, while PH and FSA are powerful methods of quantifying the differences or similarities of complex shapes, there are many considerations of such frameworks that require special attention if they are to be applied to pollen grain patterns. For example, it is not immediately apparent how to register two different pollen shapes: what portions of a spiky pollen grain surface would align with a striped one? Or how would one define an accurate measure of the difference or similarities of these shapes? Nevertheless, the space of possible patterns that pollen walls exhibit is so large that it includes many patterns seen in other biological patterned spheres. For example, icosahedral geometries found in pollen are also common to viruses (Baker et al., 1999; Dharmavaram et al., 2017), and striped and circular domains are also seen in diblock copolymer systems and lipid rafts of mono‐ and bilayer vesicles (Luo & Maibaum, 2018; Sirgist & Matthews, 2011; Zhang et al., 2014). Therefore, pollen wall patterns provide a wonderful testbed for morphometric techniques that may also describe other biological and soft matter systems.

### 3.2 Physical modeling

#### 3.2.1 Exine modeling

From imaging, staining, and genetic studies, it is now hypothesized that the callose wall acts as a rigid wall against which the primexine “spacers” push the underlying PM with a distinct wavelength to template the exine pattern (see Sections 2.4 and 2.5; Chang et al., 2012; Radja et al., 2019; Southworth & Jernstedt, 1995). Pectins are found in the divots of the PM undulations in *Arabidopsis* during the development and may contribute to the formation of these spacers. In addition, detailed imaging of the primexine shows the existence of various amphiphilic phases at different times during development. Xylans and AGPs surround probaculae in later stages of *Arabidopsis* development and perhaps contribute to these amphiphilic phases that help us to construct the exine. Unfortunately, the amounts of individual primexine components in different species are completely unknown. The distribution of other lipopolysaccharides, glycoproteins, and complex carbohydrates hypothesized to compose the primexine is also poorly understood. Nevertheless, this material is clearly “scale rich”; its components interact with each other to form patterns on scales ranging from a few nanometers up to microns. Therefore, coarse‐grained models can describe phenomena at different length scales. In coarse‐grained modeling, one circumvents the issue of poor chemical characterization by replacing atomistic detail with simplified groupings of molecules. One also performs some level of averaging of the dynamics of interactions over certain length scales such that you replace individual complicated interactions with one overall parameter. These models can elucidate universal behaviors and probe a range of parameters for polymer design and physicochemical conditions for achieving the appropriate morphology.

Beginning at the largest length scale of pattern features, it was recently identified through EM imaging that the “spacers” in the primexine are formed through a local phase separation of the primexine while mechanically coupled to the underlying cell membrane (Lavrentovich et al., 2016; Radja et al., 2019; Fitzgerald & Knox, 1995; Figure 3). This process was modeled as a phase transition from uniform (homogenous) phases to modulated (patterned) phases of coexisting dense and fluid phases using a Landau‐Ginzburg approach from statistical mechanics (Lavrentovich et al., 2016; Radja et al., 2019). This approach describes a free energy that can be minimized to identify equilibrium configurations (the patterned states; Landau & Lifshitz, 1980) and was originally used in superconducting systems and other organic or inorganic systems (Seul & Andelman, 1995) that have patterned states similar to those observed in pollen. In this model, the primexine is treated as a scalar concentration field coupled to the local cell membrane curvature. The equilibrium states of the effective free energy, found using computational methods such as simulated annealing and gradient descent, were shown to recapitulate symmetric extant pollen wall morphologies. In addition, the dynamics of a similar model identified some arrested states that recapitulate the common foam‐like or reticulate wall morphologies.

There are four main advances to this theory that would likely contribute to better predictions and applicability. First, the scalar field should be replaced with a more accurate order parameter that treats the material as liquid crystal or amphiphilic molecules. This would lead to predictions of more nuanced features of the surface patterns or may produce a different dependence on phenomenological parameters. Second, it has been shown that in similar modified Landau–Ginzburg systems, fluctuations alter the surface patterns and move phase boundaries (Luo & Maibaum, 2018), so a similar analysis should be done to determine how thermal fluctuations affect the currently predicted equilibrium states. Third, the dynamics of the phase separation should be more thoroughly studied. Finally, once more of the material constituents are elucidated, the parameters of the theory should be related to relevant physicochemical or material properties such as the composition of the primexine and callose wall, and the elasticity, surface tension, and bending rigidity of the membrane. This can be achieved by comparing the theoretical phase space to the pollen morphospace parametrized by the elucidated parameters. These variations in material properties could potentially explain the diversity of patterns of different species.

The reader may also recognize similarities in the lateral phase‐separation process of pollen pattern development and lipid raft formation (discrete lipid microdomains of varying molecular identity present in the external leaflet of the PM). In fact, it is natural to treat the primexine spacers as the liquid‐ordered phases of lipid rafts. As such, we may borrow methods used in the lipid raft field and apply them to pollen pattern modeling. The phase separation process of lipid bi‐ or monolayers and the corresponding membrane shape has been extensively studied through molecular simulations (Pantelopulos et al., 2017; Risselada & Marrink, 2008; Sodt et al., 2014), Monte Carlo (MC) simulations (Amazon & Feigenson, 2014; Hirst et al., 2013; Sreeja et al., 2015), and theoretical modeling (Almeida, 2009; Putzel & Schick, 2011; Radhakrishnan & McConnell, 2005). Molecular dynamics (MD) simulations have been used recently to identify the internal structure and pattern formation in lipid vesicles; however, pollen grains are comparatively large and our understanding of molecular identities is arguably too nascent for these methods to be particularly useful. Instead, since modulated phases have been successfully reproduced by MC simulations, with the appropriate coarse graining of primexine constituents, such methods could be easily applied to pollen patterning.

At the same time, results from modeling pollen pattern formation may be applied to lipid raft studies, whereas lipid rafts are relatively transient and are small, pollen grain patterning is cross‐linked by deposition of the chemically inert sporopollenin and are larger. Therefore, they are much easier to image. General predictions for lipid rafts could be tested in pollen systems. Furthermore, similar theoretical formalisms have been used to describe the formation of virus capsids (Dharmavaram et al., 2017) and block copolymer defect structures on spherical substrates (Zhang et al., 2014). Discretized versions of the equilibrium states of patterned spherical objects have been computationally explored (Sirgist & Matthews, 2011; Zhang et al., 2014); therefore, the analytic approaches employed in pollen studies may be applied to these systems (Lavrentovich et al., 2016; Radja et al., 2019).

On a smaller length scale, detailed imaging of the primexine reveals various phases associated with amphiphilic molecules, such as micelles, cylinders, and known lyotropic liquid crystal phases (hexagonally packed cylinders and lamellar sheets on the order of submicrons; Gabarayeva & Grigorjeva, 2016; Hemsley & Gabarayeva, 2007; Figure 3). Amphiphilic molecules have both hydrophilic and lipophilic components; this allows them to self‐assemble into various 3D structures under suitable physical conditions. Lyotropic liquid crystals are liquid crystalline polymer solutions made of such amphiphilic molecules that flow like a liquid but whose molecules are ordered in a crystalline way (Neto & Salinas, 2005). In the primexine, these phases presumably develop as the concentration of primexine constituents increases or as cation concentrations increase. There is currently a breadth of imaging data that identifies various phases in a range of different plant species. These systems are ripe for modeling (Gabarayeva & Grigorjeva, 2016; Gabarayeva et al., 2020; Hemsley & Gabarayeva, 2007; Hemsley et al., 2003).

While the method choice is left up to the reader and their level of comfort with various computational methods, the following are a few general suggestions of where to begin. MD authentically replicates the system's dynamics by numerically integrating equations of motion, this method applied to primexine modeling is hindered by our lack of understanding of its molecular composition. Furthermore, efficient equilibration and sampling of equilibrium properties are hugely time consuming in systems with long chains. MC methods, on the other hand, allow one to generate a huge ensemble of states with random sampling, thereby decreasing the long relaxation times associated with MD. Potentially hybrid methods that combine MD and MC methodologies could circumvent some downfalls of MD while retaining the level of authenticity it affords. In addition, a self‐consistent field theory (SCFT)‐based software was recently developed to reduce the many‐body problem into a simplified problem of analyzing conformations of single chains in a potential field created by surrounding chains. These SCFT computations are much less expensive than competing particle‐based simulations and allow the user to easily identify order–order transitions in a phase space highly populated with local minima and saddle points. This method was explicitly developed with the aim of lowering the energy barrier to entry for new users and has pedagogical documentation that is easy to follow (Arora et al., 2016). In any of these methods, it would be most appropriate to model primexine as either some generic lipopolysaccharides and glycoproteins or to specifically start by modeling known constituents such as AGPs and xylans.

#### 3.2.2 Aperture modeling

Aperture pattern modeling is also currently very nascent since the morphogens and molecular mechanisms that contribute to aperture patterning are mostly unknown. However, recently, the first mathematical model of a reaction–diffusion system of equations based on Gierer–Meinhardt (GM) was described in an attempt to correlate biochemical and geometric parameters of pollen grains to aperture formation (Plourde et al., 2019; Figure 3). GM reaction–diffusion models generally describe the interaction of two theoretical morphogens, one acting a short‐range activator and the other as long‐range inhibitor, as typically seen in Turing pattern systems. They are represented as a system of partial differential equations in which parameters such as diffusion coefficients, decay constants, and rates of interactions are numerically explored. An examination of the steady‐state morphologies of this model applied to pollen grain apertures recapitulated the three‐stripe *Arabidopsis* aperture morphology, the four‐stripe patterns seen in *lsq* mutants (Dobritsa et al., 2011), and the newly discovered ring‐like and pore‐like morphologies of *mcr* and *dnt* mutants, respectively (see Section 2.7 for a discussion of these mutants). Using a finite‐element partial differential equation solver, simulation results indicated that that the domain size, morphogen diffusion, and decay influence the patterns produced by the morphogens. While the geometric and kinetic factors of this model were explored, the parameters are not yet tied to underlying biologically realistic processes. Such a link may be formed when we have a better biological understanding of aperture pattern formation.

It may be possible that the patterns in different species form through different molecular and/or physical mechanisms. In some species, apertures sit within the patterning of the exine, and, thus, their distribution on the surface reflects exine patterning (Figure 1, *Phlox* sp.). In these species, it should be explored whether these patterns form through entirely different mechanisms that are more closely tied with exine patterning, and if perhaps the phase‐transition theory discussed previously could apply.

## 4 CONCLUSION

Pollen wall patterning is an extraordinarily complex process that involves many macromolecular components, genes, and likely physicochemical processes. Most research on the components and roles of the primexine, PM, callose wall, and aperture‐patterning components has been limited to model systems, and it is not known if these compositions and roles differ in other species. Consequently, it would be beneficial to apply techniques commonly used to identify polysaccharides in plant cell walls, such as immunogold labeling, to a variety of pollen grain taxa. This will reveal if and how the primexine composition differs between species (Majda, 2019; Sun et al., 2013; Yi et al., 2010). In addition, developments in real‐time monitoring of the cell wall polymers would revolutionize our understanding of the cell wall patterning process (Altatouri & Geitmann, 2014). Together, this information could reveal the important physicochemical and material parameters that result in changes in the morphospace of patterns, which may be key in understanding how different patterns can form. While many hypotheses are available at the current stage of our understanding, it is important to also consider how these mutants might inform physical mechanisms of pattern formation, not just molecular ones. Finally, an effort should also be made to understand the membrane properties of pollen cells. The primexine and cell membrane are tightly coupled systems, and the properties of both likely influence the physicochemical processes of pattern formation. Similar methodologies to the ones we have discussed may be applied to other organisms with patterned cuticles. Perhaps the universality of these patterns is dependent on common physical processes and lead to a more global understanding of pattern formation processes.

## CONFLICT OF INTERESTS

The authors declare that there are no conflict of interests.

### PEER REVIEW

The peer review history for this article is available at https://publons.com/publon/10.1002/jez.b.23005.
